# Forced Resurgence and Targeting of Intracellular Uropathogenic *Escherichia coli* Reservoirs

**DOI:** 10.1371/journal.pone.0093327

**Published:** 2014-03-25

**Authors:** Matthew G. Blango, Elizabeth M. Ott, Andreja Erman, Peter Veranic, Matthew A. Mulvey

**Affiliations:** 1 Division of Microbiology and Immunology, Pathology Department, University of Utah, Salt Lake City, Utah, United States of America; 2 Institute of Cell Biology, Faculty of Medicine, University of Ljubljana, Ljublijana, Slovenia; Institut Pasteur, France

## Abstract

Intracellular quiescent reservoirs of uropathogenic *Escherichia coli* (UPEC), which can seed the bladder mucosa during the acute phase of a urinary tract infection (UTI), are protected from antibiotic treatments and are extremely difficult to eliminate. These reservoirs are a potential source for recurrent UTIs that affect millions annually. Here, using murine infection models and the bladder cell exfoliant chitosan, we demonstrate that intracellular UPEC populations shift within the stratified layers of the urothelium during the course of a UTI. Following invasion of the terminally differentiated superficial layer of epithelial cells that line the bladder lumen, UPEC can multiply and disseminate, eventually establishing reservoirs within underlying immature host cells. If given access, UPEC can invade the superficial and immature bladder cells equally well. As infected immature host cells differentiate and migrate towards the apical surface of the bladder, UPEC can reinitiate growth and discharge into the bladder lumen. By inducing the exfoliation of the superficial layers of the urothelium, chitosan stimulates rapid regenerative processes and the reactivation and efflux of quiescent intracellular UPEC reservoirs. When combined with antibiotics, chitosan treatment significantly reduces bacterial loads within the bladder and may therefore be of therapeutic value to individuals with chronic, recurrent UTIs.

## Introduction

UPEC strains are the primary cause of UTIs, which rank among the most common of both community-acquired and nosocomial infections [1]. The majority of UPEC isolates encode adhesive fibers known as type 1 pili that enable the pathogens to bind and invade the bladder urothelium [2]–[4]. The stratified layers of epithelial cells comprising the urothelium create a formidable barrier that is impermeable to many compounds, including commonly used antibiotics [5], [6]. This tissue normally has an exceptionally slow turnover rate, but when damaged the urothelium can rapidly regenerate to reestablish barrier function [5], [7]. Microscopic analyses indicate that UPEC can replicate to high levels within the cytosol of apical terminally differentiated superficial bladder epithelial cells (BECs), forming large inclusions known as intracellular bacterial communities (IBCs) [8], [9]. Superficial cells containing IBCs are eventually shed, rendering the underlying immature BECs exposed and presumably more susceptible to infection [3], [8]–[10].

The ability of UPEC to multiply intracellularly appears to be regulated, at least in part, by the differentiation status of the BECs and the arrangement of host actin filaments [10]. Within the basolateral regions of the superficial cells where F-actin is enriched, and within immature BECs that are replete with F-actin, internalized UPEC remain bound inside of late endosome-like compartments where bacterial growth is restricted. The quiescent nature of these compartmentalized bacteria, coupled with the resilient barrier function of the urothelium, enables intracellular UPEC to withstand antibiotic treatments that effectively sterilize the urine [6], [11]–[14]. These pathogens are also protected from the rinsing flow of urine and host immunosurveillance mechanisms, and may persist within the urothelium for many weeks [15], [6], [1]. It is hypothesized that the rearrangement of actin filaments within differentiating BECs can trigger the resurgence of UPEC from intracellular reservoirs, promoting the development of recurrent and chronic UTIs [10].

Here, we use the biodegradable linear polysaccharide chitosan to interrogate the location and behavior of the UPEC reservoirs within the murine bladder. Chitosan is a deacetylated derivative of chitin that is being developed for a number of industrial, agricultural and biomedical uses, including drug and vaccine delivery in animals and humans [16], [17]. When instilled into the bladders of mice via catheterization, chitosan disrupts tight junctions and causes rapid exfoliation of the superficial epithelial cells without eliciting overt signs of inflammation [18], [19]. Upon removal of chitosan, the permeability barrier function of the urothelium is restored within several hours, and the tissue is completely regenerated within about a week [19]. Based on these observations, we reasoned that chitosan might be a useful means to artificially stimulate turnover of the bladder urothelium in order to promote the resurgence of UPEC from intracellular reservoirs, thereby rendering the pathogens more susceptible to antibiotic treatments. Results presented here support this possibility, while also revealing the dynamic nature of bacterial reservoir populations within the bladder mucosa and the effects of chitosan treatment on the fitness and infectivity of UPEC.

## Materials and Methods

### Reagents

Chitosan hydrochloride, low molecular weight (FMC Corporation), was prepared as a 0.02% stock (w/v) in phosphate buffer (1.6 g/L NaCl, 0.095 g/L KH_2_PO_4_, 0.472 g/L NaHPO_4_×12⋅H_2_O; pH 4.5). All antibiotics were purchased from Sigma-Aldrich and used at the concentrations noted in the text.

### Bacterial Strains and Growth Assays

The reference UPEC cystitis isolate UTI89 has been described previously [9], [20]. Bacteria were grown from frozen stocks in either Luria-Bertani (LB) broth or modified M9 minimal medium (6 g/liter Na_2_HPO_4_, 3 g/liter KH_2_PO_4_, 1 g/liter NH_4_Cl, 0.5 g/liter NaCl, 1 mM MgSO_4_, 0.1 mM CaCl_2_, 0.1% glucose, 0.0025% nicotinic acid, 0.2% casein amino acids, and 16.5 μg/mL thiamine in H_2_O, pH) at 37°C. Growth curve analysis was performed as previously described [21] by measuring optical density at 600 nm (O.D._600_) every 30 min using a Bioscreen C instrument (Growth Curves USA). Error bars are extremely small in these experiments and were omitted for clarity.

### Biofilm Assays

*In vitro* microtiter plate-based biofilm assays were performed as previously described [21]. Briefly, UTI89 was diluted 1∶100 from overnight shaking cultures into modified M9 medium (± chitosan as indicated). Quadruplicate, 100-μl samples in 96-well pinchbar flat-bottomed polystyrene microtiter plates with lids (Nunc) were incubated for 48 h without shaking at 37°C. Sample wells were surrounded by wells containing only distilled water in order to limit evaporation. Non-adherent bacteria were then removed by washing twice with H_2_O prior to addition of crystal violet (150 μl of a 0.1% solution in water; Sigma-Aldrich). After a 10-min incubation at room temperature, the wells were rinsed twice with H_2_O and air-dried. Dimethyl sulfoxide (200 μl) was added to each well, and the plates were shaken vigorously for 15 min on an orbital shaker to solubilize the dye. Absorbance (A_562_) was measured using a Synergy HT multidetection microplate reader (BioTek Instruments, Inc.).

### Cell Culture-based Assays

UTI89 was grown at 37°C for 48 h in static LB broth to promote type 1 pilus expression [22]. The 5637 bladder epithelial cell line (ATCC, HTB-9) was grown in complete RPMI medium (supplemented with 10% fetal bovine serum, Sigma-Aldrich) and host cell adherence, invasion and intracellular survival assays were carried out as previously described [23], [24], [25], [26]. Triplicate sets of confluent BEC monolayers in 24-well tissue culture plates were infected with UTI89 using a multiplicity of infection of ∼15 bacteria per host cell. New vials of 5637 BEC cultures are thawed every 2–3 months, and the cultures are confirmed to lack mycoplasma contamination yearly using commercially available kits. Chitosan (0.0002% or 0.002% w/v) or an equivalent volume of phosphate buffer (pH 4.5) was added with the bacteria. To facilitate and synchronize bacterial contact with the host cells, plates were centrifuged at 600×*g* for 5 min at room temperature using a Beckman Allegra 6 Centrifuge. After a 2-h incubation at 37°C, medium was collected and titered, and bladder cell monolayers were washed three times with PBS containing Ca^2+^ and Mg^2+^ (PBS^2+^) to remove any non-adherent bacteria. At this point, numbers of total host cell-associated bacteria were determined by lysing the host cells in 1 mL PBS with 0.4% Triton X-100, and plating serial dilutions of the lysates on LB agar plates. Alternatively, complete RPMI medium plus gentamicin (100 μg/mL) was added and incubations were continued for another 2 h in order to kill any extracellular bacteria. These samples were then washed with PBS^2+^ and lysed, or incubations were continued for another 14 h in RPMI medium containing a lower concentration of gentamicin (10 μg/ml) prior to final washes and host cell lysis. A submaximal concentration of gentamicin was used with the longer incubations to prevent extracellular growth of UPEC while also limiting possible leaching of the antibiotic into the host cells. The numbers of intracellular bacteria present after the 2- and 14-h gentamicin treatments were determined by dilution plating of lysates. Invasion indices were calculated by dividing the numbers of intracellular bacteria detected after the 2-h gentamicin treatment by the number of cell-associated bacteria present prior to addition of gentamicin. The numbers of surviving intracellular bacteria recovered from BECs after the 14-h incubation with gentamicin were normalized by dividing by the numbers of invading bacteria present after the 2-h incubation with gentamicin. Of note, chitosan treatments did not affect the adherence or viability of the BECs during the course of these assays, as determined by trypan blue exclusion assays (Thermo Fisher) and microscopy.

### Chitosan Treatment and Infection of Mice

Seven- to 8-week-old female CBA/J mice (Jackson Laboratory), selected at random, were anesthetized via isofluorane inhalation and inoculated slowly via transurethral catheterization with 50 μl of a bacterial suspension (∼10^7^ CFU from 24 h static LB broth cultures of UTI89) in PBS, as previously described [3]. Bacterial reflux into the kidneys using this procedure is rare, occurring in less than 1% of the test animals (unpublished observations). Chitosan (0.01% suspension in 50 μl phosphate buffer, pH 4.5) was administered via transurethral catheterization 4 h prior to the introduction of UTI89 or at days 3 and 14 after bacterial inoculation. The concentration of chitosan used in these assays reproducibly triggers the exfoliation of nearly all of the superficial bladder cells within 20 min [19]. Following injections with chitosan, mice were positioned on their sides under continued anesthesia within warmed chambers for 20 min. Midway through this incubation, the animals were flipped to their other side to ensure more uniform contact of chitosan with lumenal bladder surfaces. Bladders were then washed twice with 50 μl PBS administered via catheterization and expressed by gentle palpation. At the indicated time points, mice were sacrificed, bladders were harvested aseptically, weighed, and homogenized in 1 mL PBS containing 0.025% Triton X-100. To enumerate numbers of internalized bacteria, we employed *ex vivo* gentamicin protection assays in which isolated bladders were quartered and incubated for 1 h at 37°C in 1 mL PBS containing 100 μg gentamicin prior to rinses in PBS and subsequent homogenization [3], [27]. This *ex vivo* treatment is sufficient to kill nearly all extracellular bacteria present in the samples. Bacterial titers within the homogenates were determined by plating serial dilutions on LB agar plates. Eleven mice total, from two independent experiments, were examined for each variable tested. As these experiments are inherently variable, all CFU values, including statistical outliers, were included in the final analysis. Previous work demonstrated that mock-infected female mice co-housed with infected females do not acquire UTI [6]. The limit of quantification in these assays is considered to be 20 CFU counted on the lowest serial dilution plate, corresponding to about 200–600 CFU/g bladder.

### Antibiotic Treatments

At 3 d post-inoculation of mice with UTI89 via transurethral catheterization, animals were treated with chitosan or phosphate buffer alone. Antibiotics were administered at this time with additional doses given daily for a total of 3 or 7 consecutive days, as indicated. Gentamicin (200 μg) was injected subcutaneously, while sparfloxacin (700 μg) and ciprofloxacin (40 μg) were given orally by gavage. All antibiotics were delivered in 50 μl volumes, dissolved in water. Control animals were given water alone by oral gavage. Mice were sacrificed 3 d after the final antibiotic treatment, at which point bladders were harvested aseptically, weighed, and homogenized as described above. Antibiotic concentrations used in these assays are based on typical doses given to human patients, scaled down for use in mice [28]. All of the antibiotics employed effectively sterilize the urine of infected mice, reaching levels in the urine within 2 h post-administration that are well above the minimal inhibitory concentrations for free-living UTI89 [6]. No antibiotics were found to be especially effective against intracellular UTI89 present within the urothelium [6].

### SEM Microscopy

Infected mouse bladders and uninfected controls were excised from animals, bisected longitudinally, splayed, and pinned down lumenal side up in PBS on silicon disks (Sylgard 184 silicone elastomer, Dow Corning Corp.). Tissues were then fixed for 4 h at 4°C in 0.1 M cacodylate buffer (pH 7.4) containing 4% paraformaldehyde and 2% glutaraldehyde. Tissue samples were rinsed in 0.1 M cacodylate buffer and post-fixed in 1% osmium tetroxide in the same buffer for 1 h at 4°C. Specimens were critical point dried, sputter-coated with gold and examined at 15 kV with a JOEL JSM 84A scanning electron microscope.

### Fluorescence Microscopy

To visualize lumenal host cell nuclei, bladders from mice were bisected longitudinally, splayed, pinned down lumenal side up in PBS, fixed for 20 min at room temperature in 3.7% paraformaldehyde dissolved in PBS, rinsed with PBS 3X, and then stained for 10 min at 4°C in visualization buffer (PBS containing 0.002% saponin and 2 μg/ml Hoechst dye; Sigma-Aldrich). Following additional washes with PBS, bladders were imaged using 4X or 10X objectives on an Olympus BX51 fluorescence microscope equipped with a QImaging QIClick Cooled CCD camera.

For immunostaining, infected mouse bladders were excised from animals, bisected longitudinally, splayed and fixed for 2 h at 4°C in EM grade paraformaldehyde (PFA, Electron Microscopy Services). Samples were blocked overnight at 4°C in blocking buffer (0.2% saponin, 3% BSA, and 0.5% nonfat dry milk powder in PBS) and then incubated with goat anti-*E. coli* antibody (1∶400; BioDesign International) for 2 h at RT in antibody buffer (PBS supplemented with 0.02% saponin and 1% BSA). Following washes with PBS, bladders were incubated for 1 h at room temperature in antibody buffer containing Alexa Fluor488-conjugated donkey anti-goat secondary antibody (1∶400; Life Technologies) and Alexa Fluor546-conjugated phalloidin (1∶40; Life Technologies). Bladders were mounted in FluorSave Reagent (Calbiochem) and images were collected and processed using an Olympus FV1000XY confocal microscope and Imaris software.

### Statistical Analysis

*P* values were determined by two-tailed Student’s *t*-tests or Mann-Whitney U tests performed using Prism 5.01 software (GraphPad Software). Student’s *t*-tests were performed with Welch’s correction for unequal variances in the biofilm assays. All mouse experiments were performed using a minimum of 11 mice as required for the Mann-Whitney U test (GraphPad Software). Data distribution normality (Gaussian) was not assumed, such that non-parametric tests were used for the mouse experiments. Sample sizes are consistent with those from similar studies. Values of less than 0.05 were defined as significant for all experiments.

### Ethics Statement

Mice used in this study were handled in accordance with protocols approved by the Institutional Animal Care and Use Committee at the University of Utah (Protocol number 10-02014), following US federal guidelines indicated by the Office of Laboratory Animal Welfare (OLAW) and described in the Guide for the Care and Use of Laboratory Animals, 8th Edition.

## Results and Discussion

**Figure 1:**
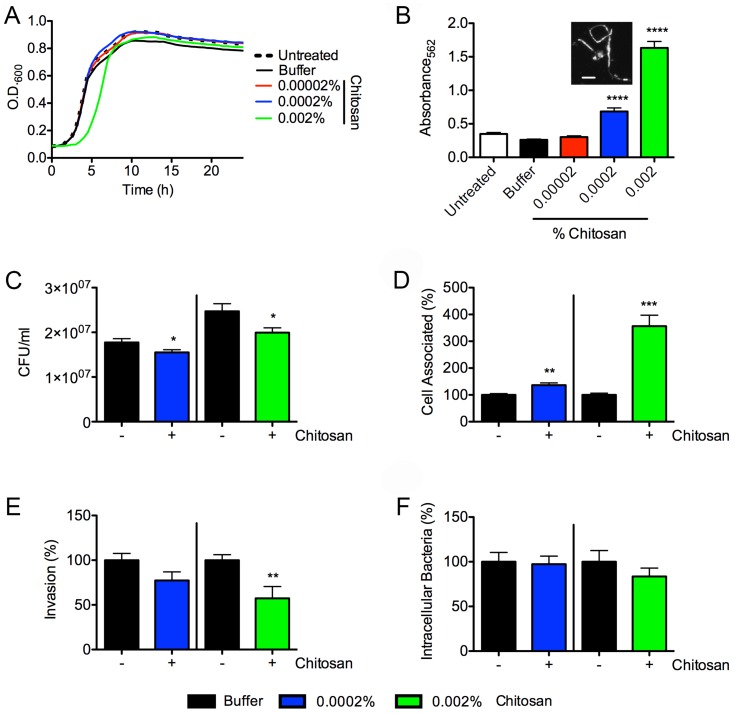
Chitosan affects UPEC growth, biofilm formation, and interactions with BECs. (**A**) Growth of UTI89 in modified M9 medium containing increasing concentrations of chitosan (0.00002, 0.0002, 0.002%) or phosphate buffer (pH 4.5). Data shown are representative of three independent experiments carried out in triplicate. (**B**) Graph shows chitosan effects on biofilm production by UTI89 relative to untreated controls, as measured by microtiter plate assays. Data represent mean results ± SEM from three separate experiments performed in quadruplicate. Inset shows an example of filamentous bacteria in 0.002% chitosan; scale bar, 10 μm. Chitosan (0.0002 or 0.002%) effects on (**C**) UTI89 growth in RPMI medium during a 2-h incubation with 5637 BECs, (**D**) bacterial attachment to BECs, (**E**) bacterial invasion of BECs, and (**F**) bacterial survival within BECs over a 14-h period were quantified relative to buffer-treated controls. The data in **F** were normalized by dividing the numbers of surviving intracellular bacteria recovered from BECs after the 14-h incubation with gentamicin by the numbers of invading bacteria present after the 2-h incubation with gentamicin. Graphs show mean results ± SEM from three independent experiments performed in triplicate. *P<0.05, **P<0.01, ***P<0.001 versus buffer-treated controls, as determined by Student’s *t* test with Welch’s correction when appropriate.

Previous work indicated that at high concentrations chitosan could disrupt bacterial cell membranes and rapidly kill *E. coli* [29]. In assays using the UPEC reference strain UTI89 grown shaking in modified M9 minimal medium containing 0.002, 0.0002, or 0.00002% chitosan or carrier alone (phosphate buffer, pH 4.5), we found that logarithmic growth of the pathogen was delayed by the higher concentration of chitosan, but otherwise normal (Fig. 1A). In microtiter plate-based assays using static cultures, chitosan enhanced the development of bacterial communities known as biofilms in a dose-dependent fashion (Fig. 1B). Environmental stresses can accentuate biofilm formation [30], which in turn may promote UPEC persistence within the urinary tract [31]. Interestingly, the higher concentrations of chitosan used in our assays notably increased the numbers microscopically observed filamentous bacteria (Fig. 1B, inset), an indicator that chitosan may activate stress response pathways within UPEC.

Potential effects of chitosan on interactions between UTI89 and BECs were examined using the human bladder cell line designated 5637. Chitosan (0.0002 or 0.002%) did not alter the attachment or viability of the cultured BECs, but did have a modest, though statistically significant, inhibitory effect on bacterial growth within the cell culture medium (Fig. 1C). In contrast, UPEC adherence to the BECs was increased up to 4-fold in the presence of chitosan (Fig. 1D), but this did not lead to coordinate increases in host cell invasion frequencies as determined by gentamicin protection assays. Instead, the higher concentration of chitosan used (0.002%) somewhat attenuated bacterial entry into the BECs (Fig. 1E). The chitosan treatments had no net effect on the numbers of intracellular bacteria recovered from the cultured BECs after a 14-h incubation in which extracellular growth of UTI89 was prevented due to the continued presence of the host cell impermeable aminoglycoside antibiotic gentamicin (Fig. 1F). In total, these *in vitro* results indicate that chitosan has at best only slight effects on the growth of UPEC in cell culture medium, while at the same time it markedly enhances bacterial adherence to the host cells. However, the bound bacteria are somewhat less invasive in the presence of chitosan, possibly because chitosan induces bacterial filamentation, which in turn may hinder UPEC entry into host cells [32].

**Figure 2:**
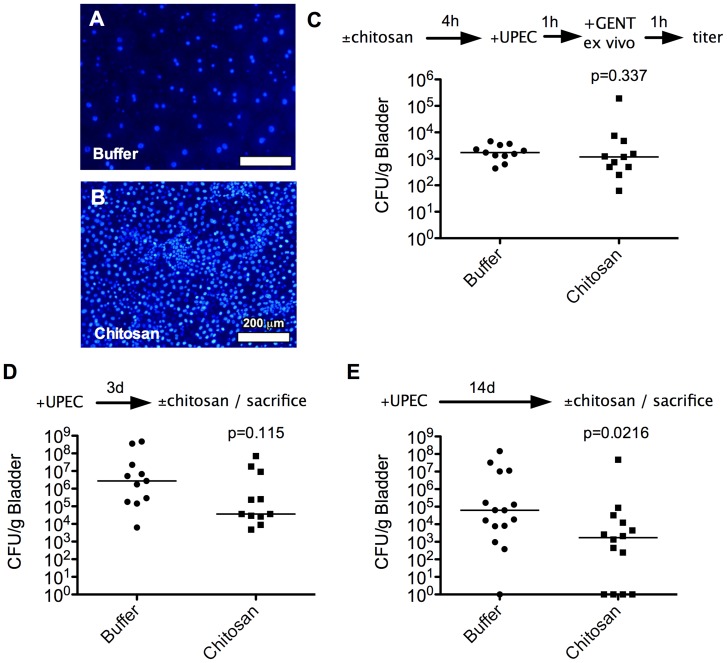
Chitosan-induced exfoliation of superficial BECs and analysis of UPEC reservoir populations. (**A** and **B**) Fluorescent images show the lumenal surfaces of mouse bladders fixed and stained with Hoechst dye following 20-min treatments with (**A**) phosphate buffer (pH 4.5) alone or with (**B**) 0.01% chitosan. Representative images of three independent experiments performed in duplicate. (**C**) The bladders of adult female CBA/J mice were inoculated with UTI89 via transurethral catheterization 4 h after a 20-min treatment of the mouse bladders with chitosan or phosphate buffer alone. Internalized bacteria were enumerated 1 h after initiation of the infection using *ex vivo* gentamicin protection assays. (**D** and **E**) Graphs show bacterial titers present in the bladders of mice treated with phosphate buffer or chitosan just before collection at (**D**) 3 d or (**E**) 14 d post-inoculation with UTI89. Bars indicate median values for each group; n = 11 mice. *P* values indicated were determined by the Mann-Whitney U-test.

To assess the effects of chitosan on UTI *in vivo*, we used a model system employing adult female CBA/J mice. Instillation of chitosan into the bladder lumen via transurethral catheterization induced exfoliation of nearly all of the large, typically binucleate superficial cells within 20 min, whereas bladders treated with phosphate buffer (carrier) alone remained intact (Fig. 2A–B). These results are in line with earlier observations [19], and provided an opportunity to quantify the ability of UPEC to directly invade immature bladder cells within the urothelium. Although previous microscopy-based work indicated that UPEC could invade all layers of the bladder urothelium, UPEC entry into the immature cells appeared to be infrequent [10]. To determine if immature cells within the urothelium are inherently resistant to invasion by UPEC or simply less accessible, mouse bladders were treated with chitosan or buffer alone, washed with PBS, and allowed to recover for 4 h prior to inoculation with UTI89 via catheterization. After 1 h, bladders were collected, quartered, and incubated for an additional hour in the presence of gentamicin to kill any extracellular microbes. Titration of surviving bacteria within the bladders indicated that UTI89 could invade both superficial and exposed immature bladder epithelial cells at similar levels (Fig. 2C).

We next asked if UPEC could persist within both the mature superficial and immature BECs. Mice were inoculated with UTI89 via catheterization and then returned to their cages for 3 or 14 d, allowing time for the establishment of UPEC reservoirs [6], [14], [33]. Just prior to recovery, bladders were treated with buffer alone or with chitosan to remove superficial cells. At the 3 d time point, chitosan treatment led to a discrete, though statistically insignificant, decrease in bacterial titers within the bladder (Fig. 2D). By 14 d, chitosan treatment of the bladder just before collection caused a more discernable, statistically significant reduction in bacterial numbers (Fig. 2E). These results indicate that UPEC can establish reservoirs within both the superficial and underlying immature layers of the urothelium, and that these reservoirs can shift apically towards the lumenal surface over time.

**Figure 3:**
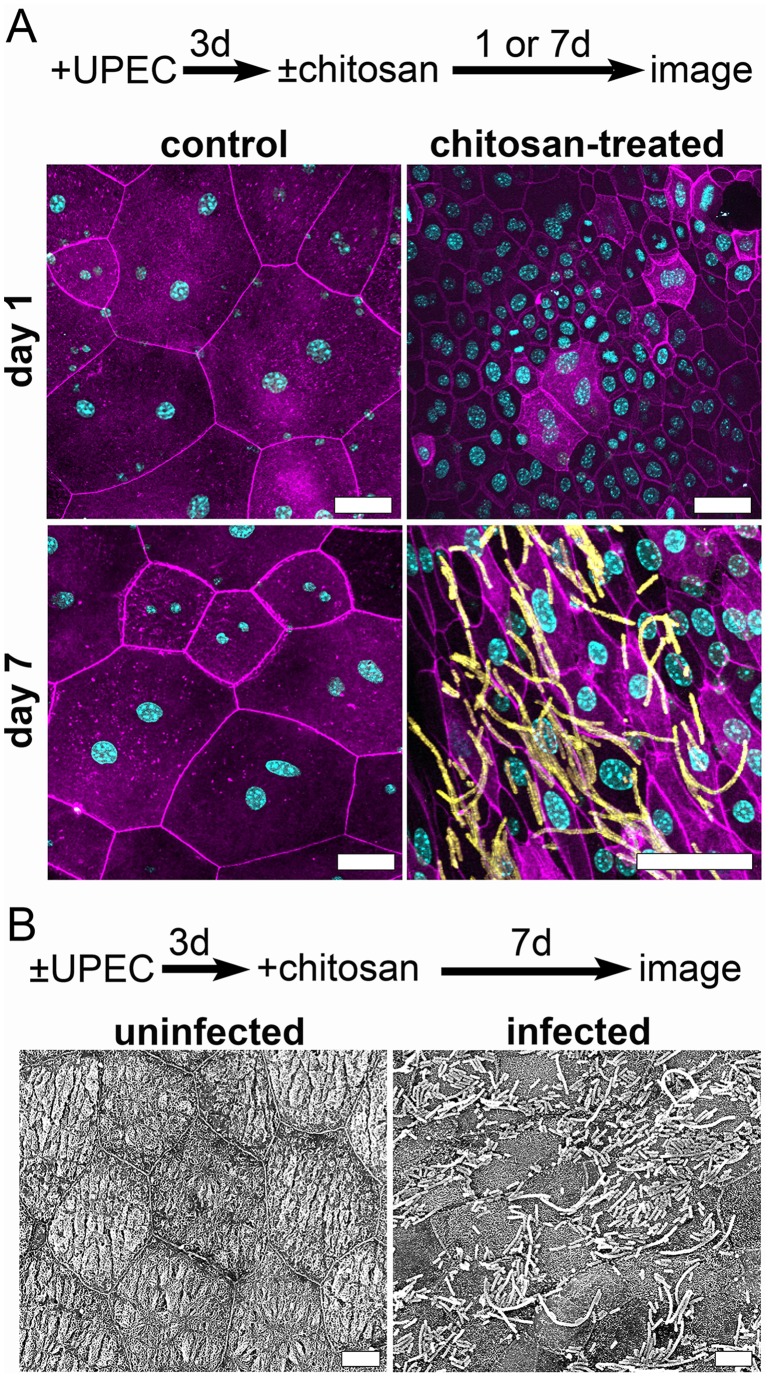
Chitosan treatment promotes the resurgence of UPEC reservoir populations into the bladder lumen. Adult female CBA/J mice were inoculated with UTI89 via transurethral catheterization or left uninfected. After 3 d, phosphate buffer alone (control) or 0.01% chitosan was instilled into the bladders for 20 min prior to rinses with PBS. Bladders were bisected, splayed and imaged 1 or 7 d later, as indicated, using (**A**) confocal fluorescence microscopy or (**B**) SEM. Images in (**B**) show F-actin (purple), host nuclei (blue), and bacteria (yellow). Scale bars, (**A**) 50 μm and (**B**) 10 μm. Images are representative of three independent experiments.

The exfoliation of BECs stimulated by chitosan treatment triggers regenerative processes in the urothelium, culminating in terminal differentiation of new superficial cells with coordinate redistribution of the actin cytoskeleton [19], [34]. The possibility that these events provide an environment that favors the resurgence of intracellular UPEC reservoirs was investigated by imaging mouse bladders at 1, 3 or 7 d post-treatment with chitosan. As determined by both fluorescence confocal and scanning electron microscopy, the instillation of chitosan into mouse bladders 3 d after inoculation with UTI89 often led to the appearance of numerous, surface-localized bacteria within the week following chitosan treatment (Fig. 3). Many of the bacteria observed 7 d after the brief 20-min treatment of the bladders with chitosan were filamentous, similar to those seen emerging from infected BECs in mice during the acute phase of experimental UTI [8], [33]. In general, few bacteria were observed on the lumenal surfaces of bladders recovered 1 or 3 d after chitosan treatment, before extensive terminal differentiation of the regenerating urothelium takes place (Fig. 3A and unpublished observations). No bacteria were seen in association with uninfected bladders regardless of chitosan treatment (Fig. 3B), and few, if any, bacteria were detected on the surfaces of infected bladders treated with phosphate buffer alone (Fig. 3A). These observations suggest that chitosan can stimulate the resurgent growth and efflux of UPEC from established intracellular reservoirs by triggering the exfoliation of superficial cells and subsequent differentiation of the underlying BECs.

**Figure 4:**
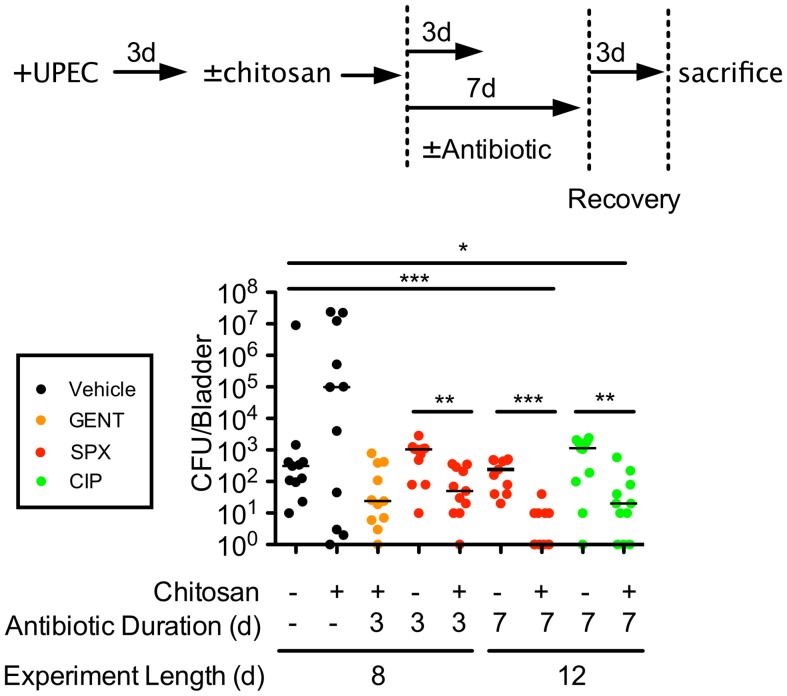
Chitosan treatment enhances the efficacy of antibiotics in reducing bacterial titers within the bladder. Mice were infected with UTI89 via transurethral catheterization 3% chitosan. After a 20-min incubation and subsequent washes with PBS, mice were treated for 3 or 7 d with gentamicin (GENT), sparfloxacin (SPX), or ciprofloxacin (CIP), as indicated. Mice were sacrificed 3 d after cessation of antibiotics or mock treatments. An outline of the experimental setup is shown on top. Bars in the graph indicate median values for each sample group; n = 11 mice total from two independent assays. *P<0.02, **P<0.005, ***P≤0.0002; as determined by Mann-Whitney U-tests.

By forcing the resurgence of UPEC from intracellular reservoirs, we reasoned that chitosan may render the pathogens more susceptible to host defenses and antibiotics. To assess this possibility, mice were treated with chitosan or buffer alone at 3 d post-inoculation with UTI89 followed by the administration of 3 or 7 daily doses of antibiotics. After an additional 3 d to allow the animals to clear the antibiotics, bladders were recovered, homogenized and plated to determine bacterial titers. The antibiotics used in these assays included gentamicin and the host cell-permeable fluoroquinolones ciprofloxacin and sparfloxacin (Zagam). Both ciprofloxacin and sparfloxacin effectively kill intracellular UPEC in cell culture-based assays, but are mostly ineffective against persistent intracellular bacterial populations in the mouse UTI model [6]. Chitosan treatment without coordinate administration of antibiotics stimulated a marked outgrowth of UTI89 in the bladders of some mice, while reducing bacterial titers in others (Fig. 4). These data agree with our microscopic observations (Fig. 3), suggesting that in many mice chitosan-initiated turnover of the urothelium can promote the multiplication and reemergence of intracellular UPEC back into the bladder lumen. In a few animals in which UPEC reservoirs are already situated near the bladder surface, chitosan-induced exfoliation of the superficial BECs may on its own be an effective means to expunge bacteria from the bladder, stimulating the release of infected host cells and their subsequent clearance with the flow of urine.

The administration of gentamicin prevented the outgrowth of UPEC following chitosan treatment, but did not significantly reduce overall bacterial titers relative to untreated controls (Fig. 4). The delivery of sparfloxacin for either 3 or 7 d without prior chitosan treatment also had minimal effects on bacterial titers within the bladder in comparison with untreated controls and in line with previous observations [6]. However, the treatment of bladders with chitosan followed by the administration of sparfloxacin significantly reduced bacterial titers, an effect that was more pronounced with the 7-d antibiotic treatments (Fig. 4). Similar results were obtained using a 7-d treatment with ciprofloxacin, an antibiotic that is more widely approved for use in humans than sparfloxacin. In total, these findings show that chitosan treatment can enhance the effectiveness of antibiotics at eliminating intracellular bacterial populations that may become embedded within the urothelium during the course of a UTI.

**Figure 5:**
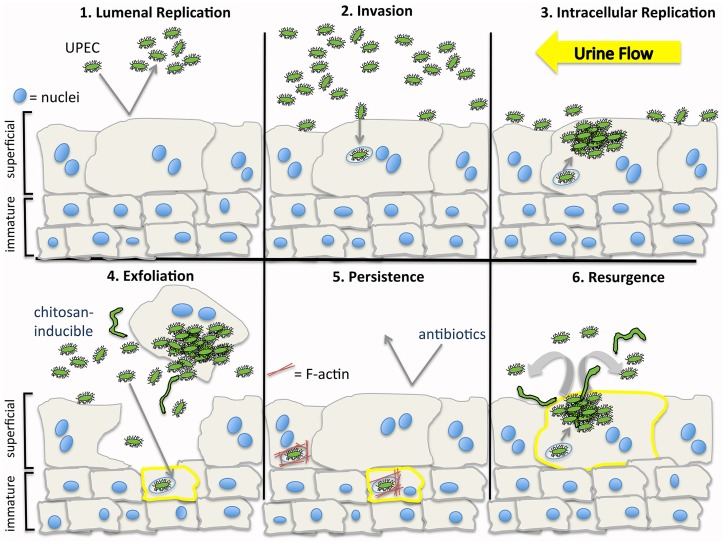
UPEC persistence and recurrence within the bladder. (**1**) UPEC that gain entry into the bladder lumen can multiply within the urine and in association with the bladder surface. (**2**) Some bacteria enter host superficial cells and are trafficked into late endosome-like compartments. (**3**) Shearing forces from the flow of urine, secreted antimicrobial factors, and infiltrating neutrophils can eliminate many bacteria, while internalized UPEC are sheltered. Some intracellular bacteria may enter the host cytosol where they can rapidly multiply, forming IBCs. (**4**) Infection can induce the exfoliation of the superficial cells, exposing underlying immature BECs (**5**) that can rapidly differentiate to reestablish barrier function. Chitosan treatment can also stimulate exfoliation of superficial bladder cells and subsequent regenerative processes. Within both the superficial and immature cells of the bladder, UPEC can persist as quiescent reservoirs within membrane-bound compartments enmeshed in F-actin. Antibiotics that can effectively sterilize the urine are ineffective against the intracellular UPEC reservoirs. (**6**) Redistribution of actin and perhaps other signals associated with terminal differentiation of the BECs may elicit the resurgent growth of UPEC, resulting in bacterial release back into the bladder lumen. By promoting turnover of the urothelium, the intravesical delivery of chitosan can stimulate the resurgence of UPEC from intracellular reservoirs, making the bacteria more accessible to antibiotics.

The idea of artificially stimulating bladder cell exfoliation as a therapeutic option for the treatment of chronic and/or recurrent UTIs has been explored experimentally in previous work using protamine sulfate [35], [36]. When instilled into the bladder lumen, protamine sulfate strips away layers of the urothelium, seriously compromising the urothelial barrier and inducing notable inflammatory responses [37]. Chitosan offers a more restrained alternative, acting primarily on the superficial cell layer and reducing the urothelial barrier function for only a short time while inducing little, if any, inflammation [19], [38]. Using chitosan as a tool, we found that UPEC can effectively invade all layers of the urothelium and, in separate experiments, demonstrated that intracellular UPEC reservoirs established within immature BECs can migrate towards the bladder lumen where they can eventually multiply and resurface (Figure 5). Our data indicate that the ability of chitosan to induce remodeling of the urothelium triggers the resurgence of intracellular UPEC reservoirs, making the bacteria more susceptible to antibiotic treatments. Emerging studies with human patients suggest that the ability of UPEC to invade BECs and persist intracellularly is not restricted to the mouse urinary tract [39]–[41], raising the possibility that the optimized use of chitosan or other bladder cell exfoliants in combination with antibiotics may be of therapeutic value for the treatment of recalcitrant UTIs in some human patients.
